# S100 Calcium-Binding Protein B and Glial Fibrillary Acidic Protein in Patients with Mild Traumatic Brain Injury

**DOI:** 10.30476/BEAT.2021.89355.1231

**Published:** 2021-10

**Authors:** Ali Meshkini, Amir Ghorbani Haghjo, Zahra Hasanpour Segherlou, Masoud Nouri-Vaskeh

**Affiliations:** 1 *Road Injuries Prevention Research Center, Tabriz University of Medical Sciences, Tabriz, Iran*; 2 *Biotechnology Research Center, Tabriz University of Medical Sciences, Tabriz, Iran*; 3 *Medical Philosophy and History Research Center, Tabriz University of Medical Sciences, Tabriz, Iran*

**Keywords:** Glial fibrillary acidic protein, S100B, Computed Tomography scang, Traumatic brain injury

## Abstract

**Objective:**

To examine the GFAP and S100B ability in prevention unnecessary brain Computed tomography (CT) scan in mild traumatic brain injury (mTBI) and compare them with the single extremity fracture in orthopedic patients.

**Methods:**

In this prospective cohort study, two orthopedics patients’ groups and mTBI patients were studied to assess the biomarkers’ ability in prevention unnecessary brain CT scan at the emergency setting. There were 40 orthopedics’ patients with single extremity fracture and 41 mTBI patients. Brain CT scans were done for all mTBI patients.

**Results:**

Brain CT scans showed no intracranial traumatic lesions. The median levels for S100B in the mTBI group was 14.8 (4.4-335.9) ng/L, and in orthopedic patients’ group was 13.3 (5-353.10) ng/L. Statistically significant differences were observed between both groups in S100B levels (*p*=0.006). The median Glial Fibrillary Acidic Protein (GFAP) levels in the mTBI patients’ group were 600 (400-16300) and in the orthopedic patients’ groups was 60 ng/L (300-14900). Statistically significant differences were observed between groups in GFAP (*p*=0.041).

**Conclusion:**

Our results showed that S100B and GFAP serum levels were significantly higher in patients with mTBI than in patients with a single limb fracture.

## Introduction

According to the Glasgow Coma Scale (GCS) score, traumatic brain injury (TBI) is categorized into mild, moderate and severe TBI. mild traumatic brain injury (mTBI) is a traumatic force resulting in brain dysfunction. It is a temporal condition; however, it may cause a long-lasting outcome [1, 2]. mTBI potential risks are intracranial hemorrhage and diffuse axonal injury and it increases the risks for impairment in psychosocial, cognitive and physical activity and in the future [3-6]. In emergency departments, mTBI diagnosis is based on physical examination, brain computed tomography (CT) scan and in some cases, brain magnetic resonance imaging (MRI). CT scan exposes people to radiation and it has low sensitivity in some cases. On the other hand, brain CT images are normal in most cases and patients are discharged from hospital with simple treatments. Unlike other organs in the brain field, we do not have any Food and Drug Administration (FDA) approved biomarker that makes mTBI diagnosis easier. Therefore, many studies have been done to explore suitable biomarker in the last years [7]. 

S100B is an intracellular calcium regulator protein in astrocytes that binds calcium with low affinity. When astrocytes die, S100B comes out of cells [8]. Most of the researches is about S100B and its relationship with brain CT’s findings [9-14]. Some issues should be considered while using S100B as a neurotrauma biomarker; for example, it is increased in general trauma patients as well. Another problem is that it is not specific to astrocytes and other cells that have S100B are chondrocytes, melanocytes and adipocytes. Glial Fibrillary Acidic Protein (GFAP) is another biomarker that has been studied as the most biomarker in TBI patients. It is a monomeric intermediate protein that was isolated first by Eng *et al*. and exists in the astroglial skeleton in both white and gray brain matter [15]. It has been shown that higher GFAP is correlated with positive brain CT findings in mTBI patients. And it has been shown that GFAP is more specific to brain injury and it is rarely affected by orthopedic injury. Our study aimed to examine the GFAP and S100B ability in prevention unnecessary brain CT in mTBI and compare them with the single extremity fracture in orthopedic patients. In this study, we hypothesized that there is no relationship between these serum biomarkers and mTBI CT scan brain findings. 

## Materials and Methods

This prospective cohort study was conducted on 81 patients (41 adults with mTBI and GCS score of 13 to 15, and 40 non-TBI orthopedic patients) who refer to the hospital up to 6 hours after trauma. The study was performed in the emergency department of two Level I trauma centers tertiary hospitals in the Northwest of Iran. This study was approved by the Medical Ethics Committee of Tabriz University of Medical Sciences. The written informed consent was obtained from the patients and/or their legally authorized representatives before the enrolment. According to the medical history, patients who enrolled in this study presence the blunt head trauma and its consequences: either loss of consciousness, amnesia, or disorientation and presenting to ER less than 6 hours of injury and physical examination (GCS score=13-15). Emergency department physicians already have had done brain CT for all patients. Exclusion criteria were the neurodegenerative disorders presence or other active central nervous system disorders, the presence of known cancer, or the presence of general trauma. Inclusion criteria for the group of orthopedic patients were the single extremity fracture presence, a GCS score of 15, and referring to the emergency department before 6 hours following an injury, whereas the exclusion criteria were head trauma, a GCS score of less than 15, neurodegenerative disorders or other active central nervous system disorders, cancer and general trauma.

A 2 mL peripheral blood sample were taken from both groups. We centrifuged the blood within the first minutes and the separated serum was placed in bar-coded containers and immediately transported to the central laboratory. Serum GFAP and S100B levels were measured by using an enzyme-linked immunosorbent assay kit. Appropriate treatment was considered by their physicians in emergency department for patients and the research team was not participate in decisions-making process. Orthopedic patients underwent usual x-ray based on the trauma site and as mentioned prior they were included after approving a single fracture in their extremities and after checking for the exclusion criteria (same as the mTBI patients).

The study outcomes included the intracranial traumatic lesions presence or absence on initial CT scans in the mTBI group and presence of extremity fracture on initial x-ray in orthopedic patients, and the lab results (the measurement S100B and GFAP serum levels). 

Descriptive statistical methods were used to express statistical information. Mean±SD was used for quantitative variables and frequency and percentage were applied for qualitative variables. Data were analyzed by the IBM SPSS software, version 16. Biomarker levels were continuous data, were expressed in ng/L, and assessed as median±SD. According to the group’s gender, group comparisons and age were performed. The normal data distribution was evaluated by the Kolmogorov-Smirnov test. The Chi-square test was used to compare qualitative data and the correlation Pearson test was employed to compare quantitative data. A *p*-value of less than 0.05 was considered significant in all cases. We calculated the sample size based on previous studies of GFAP and S100B [13, 16-21]. Serum concentrations of S100B were measured by a human S100B enzyme-linked immunosorbent assay (ELISA) kit (Bioassay Technology Laboratory, China, Catalog No: E3669Hu) using an ELISA plate reader (STATFAX-2100, Multidetector Multi-Plate Reader, USA). The detection range of the S100B ELISA kit was 0.5 ng/L-300 ng/L. GFAP concentration was also determined by ELISA kit (Bioassay Technology Laboratory, China, Catalog No: E2094Hu) with 0.05 ng/L-15 ng/L detection range. We expressed the ranges in nanogram per liter (ng/L) for coordination with previous studies. We considered the cutoff> 100 ng/L for both biomarkers as abnormal that could have been a positive predictor of the brain CT scan. Considering the significant time interval from the time that samples were taken to the tests (at least one month), the tests were not available at the patient’s bedside and did not affect the treatment process. 

All CT scans of the case group were examined in the emergency room by the clinicians and appropriate treatment was done based on CT scan results. All the CT scans were reviewed by a neurosurgeon who was not informed about the patient’s clinical information (except for sex and age) and the lab results. All CT scans results were normal.

The x-rays and CT scans of the extremity related to the control group were reported by the orthopedist and appropriate treatments were considered accordingly. The cutoff points for S100B and GFAP were derived from similar articles to triage patients requiring brain CT scan and detect possible intracranial lesions in CT scans.

## Results

**Fig. 1:**
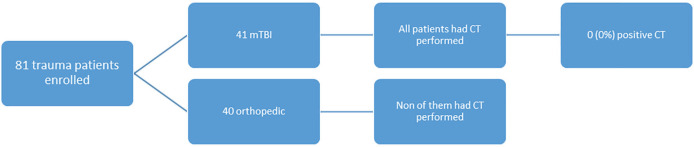
Forty orthopedics’ patients and 41 mild traumatic brain injury (mTBI) patients were enrolled into study, none one these patients had positive brain brain computed tomography (CT) lesion

Of 81 mTBI patients, 41 patients had mTBI and 40 had a single extremity (limb) fracture. Brain CT scan was performed in 41 mTBI patients and none of them had traumatic intracranial lesions on CT. Single limb fracture was the eligibility for enrollment in the control group, therefore, all of them had a single limb fracture in their x-rays (Figure 1). 

The patient’s median age was 38±19 years (33±14 years in the case group and 45±22 years in the control group). Also, 71.6 % of them were male (73% in the case group and 70% in the control group). No statistically significant differences were observed in the age, gender and race between the two groups. In the mTBI group, all of the patients had a GCS of 15; and the most common injury cause was a vehicle accident (60%). In the mTBI group it has been shown that there is no relationship between age and gender and serum S100B (respectively: *p*=0.257; *p*=0.400). It has been shown there was no relationship between age and gender and serum GFAP (respectively: *p*=0.129; *p*=0.222) in the mTBI group. Similar to the orthopedic patients, the most common injury cause was vehicle accidents (82%). In the control group it has been shown that there was no relationship between age and gender and serum S100B (respectively: *p*=0.385; *p*=0.862). In the control group, it has been shown that there was no relationship between age and gender and serum GFAP (respectively: *p*=0.635; *p*=0.766). 

The median S100B in the mTBI group was 14.8 (4.4-355.9) ng/L; whereas it was 13.3 (5-353.10) ng/L in orthopedic patients. Statistically significant differences were between groups for S100B levels (*p*=0.006). The median GFAP levels in the mTBI patients’ group were 600 (300-14900) ng/L and in the orthopedic patients’ group was 600 (400-16300). The GFAP levels were significant differences between the groups (*p*=0.041) (Table 1). 

**Table 1:** Mild traumatic brain injury (mTBI) frequency and orthopedics patients in each biomarker group

	**S100B** ^c^ **>100 ng/L**	**S100B** **<100 ng/L**	**GFAP** ^a^ **>100 ng/L**	**GFAP** **<100 ng/L**	**GFAP** ** >6000 ng/L**	**GFAP ** **<6000 ng/L**
mTBI^b^ patients	7	34	41	0	6	33
Orthopedics patients	2	38	40	0	40	0

^a^GFAP: Glial Fibrillary Acidic Protein; ^b^mTBI: Mild Traumatic Brain Injury; ^c^S100B: *S100 Calcium-binding Protein B*

In our study, unfortunately, there was no positive CT scans to compare with negative ones. The cutoff points were derived from similar articles to triage patients requiring brain CT scanning for S100B and GFAP and detect possible intracranial lesions in CT scans. As we did not have any positive CT scan, we expected all of the case group would have serum biomarkers beneath cutoff. Considering 100 ng/L as a cutoff point for both biomarkers, in the mTBI group, 82.9% of the patients had the S100B serum level below the cutoff point and 00.0% of the patients had a GFAP serum level below the cutoff point. However, at a cutoff point of 6000 ng/L for GFAP, S100B showed similar results (6 patients, 85%). In the orthopedics group, two patients had the S100B level above the cutoff point and all patients had a GFAP level above the cutoff point (95% and 00.0%, respectively).

## Discussion

This prospective cohort study compared the GFAP and S100B serum level and brain CT scans findings in mTBI and orthopedic patients presenting to a Level I trauma center.

According to previous studies, we considered a 100 ng/L cutoff point for both biomarkers that can be an indicator of a positive brain CT scan. As we did not have a positive CT scan, we did not expect a higher than 100 ng/L level. In our study, 17.1% of mTBI group patients had S100B levels higher than 100ng/L; which indicates a specificity of 82.9%. However, GFAP was not as good as S100B; at 100 ng/L cutoff point of GFAP, the specificity was 00.0%. By an increasing in the cutoff point up to 6000 ng/L, six patients showed higher levels (over 6000 ng/L) - the same patients that had S100B levels higher than 100ng/L- which gives us 85% specificity. The orthopedic patients’ had a single extremity fracture and also had no history of head trauma. In orthopedic patients, two cases had S100B levels of higher than 100 ng/L. All patients in the orthopedic patients had GFAP level of more than 100 ng/L. By the cutoff level up to 6000 ng/L increase, the number of positive patients decreased to two cases: the same patients that had S100B levels of higher than 100ng/l and the specificity increased up to 95%. It shows that in the general traumas presence, both biomarkers are increased which raises the concern that when they are used alone as an indication of brain CT scans, regardless of screening for this biomarker at a patient’s bedside it may cause unnecessary brain CT scans. It should be confirmed that multiple trauma patients as well as general trauma lesions should not be used to identify patients with positive CT scans; and still, patient’s bedside is preferable to these biomarkers. 

A study conducted by Papa *et al*., [22] investigated the extracranial sources of S100B and GFAP and showed that both biomarkers increase in patients with general trauma, which is similar to our study; but their study found that S100B was more affected than GFAP in general traumas. And in general trauma patients especially in extracranial fractures, GFAP is better than S100B in finding CT intracranial lesions. But in our study with a cutoff of 100 ng/L, S100B had a clear advantage over GFAP and at a higher cutoff, GFAP (6000 ng/L) was similar to S100B. The difference between our study and Papa was that they included traumatic patients before fracture approving and they divided them into two groups after the presence or absence of fracture and demonstrated that serum levels of these biomarkers were significantly higher in patients with fracture. 

A study by Posti *et al*., [23] compared serum levels of these biomarkers in patients with mTBI and orthopedic patients and showed that the overall hospital GFAP levels in orthopedic patients were higher than those with mTBI with negative CT scans on admission. In contrast, we found that GFAP and S100B were significantly higher in the mTBI groups. However, they emphasized the importance of obtaining samples within 6 hours of mTBI (due to short half-life) and not using them in patients with extracranial lesions because the source of S100B may be from extracranial [24]. One risk that should be avoided, is that the S100B biomarker should not be used publicly without clinical indication in the presence of general trauma to identify people with positive CT; because, it increases the CT use instead of reducing the need for CT scans. Another thing to notice is that not all brain pathologies are visible on CT scans [25]. Some lesions are only visible on magnetic resonance imaging (MRI) or more accurately on diffuse tensor imaging (DTI). Some researchers suggest that some of these “false positive” S100B levels may indicate lesions that can be seen on MRI [21]. The S100B’s ability to detect intracranial lesions would be increased with the advance of more sensitive radiologic techniques. 

The relatively small sample size was the main limitation of this study and the findings should be further validated by using a larger sample size. Given that we used the ELISA method to perform the tests, which takes approximately 5-6 hours to complete. Some clinical conditions and emergencies may not be feasible in some cases, therefore, further studies are needed to evaluate other faster methods and compare them by standard ELISA method. Considering the different cutoff points in different studies, more studies are recommended to find the sensitivity and specificity of these tests. All patients had a GCS of 15, therefore, the patients’ outcomes cannot be able to generalized to those with lower GCS scores. Finally, this study did not investigate the relationship between patients’ prognosis and biomarker levels, which is suggested to be considered in future studies.

In conclusion, the results showed that serum levels of S100B and GFAP biomarkers were significantly higher in mTBI patients than in single limb fracture patients; and these biomarkers also were effective to prevent performing unnecessary brain CT scans. It was shown that S100B at a cutoff point similar to previous studies, had good specificity and it could prevent unnecessary CT scans up to 80%; however, in our study this cutoff point for GFAP was much higher. 
